# The natural dietary genistein boosts bacteriophage-mediated cancer cell killing by improving phage-targeted tumor cell transduction

**DOI:** 10.18632/oncotarget.10662

**Published:** 2016-07-18

**Authors:** Effrosyni Tsafa, Mariam Al-Bahrani, Kaoutar Bentayebi, Justyna Przystal, Keittisak Suwan, Amin Hajitou

**Affiliations:** ^1^ Phage Therapy Group, Department of Medicine, Imperial College London, Hammersmith Hospital Campus, London, United Kingdom; ^2^ Biotechnology Laboratory (Medbiotech), Medical and Pharmacy School, University Mohammed V de Rabat, Rabat, Morocco

**Keywords:** bacteriophage display, genistein, cancer therapy, isoflavone, phage therapy

## Abstract

Gene therapy has long been regarded as a promising treatment for cancer. However, cancer gene therapy is still facing the challenge of targeting gene delivery vectors specifically to tumors when administered via clinically acceptable non-invasive systemic routes (i.e. intravenous). The bacteria virus, bacteriophage (phage), represents a new generation of promising vectors in systemic gene delivery since their targeting can be achieved through phage capsid display ligands, which enable them to home to specific tumor receptors without the need to ablate any native eukaryotic tropism. We have previously reported a tumor specific bacteriophage vector named adeno-associated virus/phage, or AAVP, in which gene expression is under a recombinant human rAAV2 virus genome targeted to tumors via a ligand-directed phage capsid. However, cancer gene therapy with this tumor-targeted vector achieved variable outcomes ranging from tumor regression to no effect in both experimental and natural preclinical models. Herein, we hypothesized that combining the natural dietary genistein, with proven anticancer activity, would improve bacteriophage anticancer safe therapy. We show that combination treatment with genistein and AAVP increased targeted cancer cell killing by AAVP carrying the gene for Herpes simplex virus thymidine kinase (*HSVtk*) in 2D tissue cultures and 3D tumor spheroids. We found this increased tumor cell killing was associated with enhanced AAVP-mediated gene expression. Next, we established that genistein protects AAVP against proteasome degradation and enhances vector genome accumulation in the nucleus. Combination of genistein and phage-guided virotherapy is a safe and promising strategy that should be considered in anticancer therapy with AAVP.

## INTRODUCTION

Cancer is a major cause of mortality and morbidity worldwide despite progress in the conventional therapies and despite the fact that several mechanisms of oncogenesis are now understood. Developing efficient systemic therapies would play a major advance in cancer treatment. Indeed, most cancer patients die of metastases and systemic chemotherapy is the most widely used treatment for cancer. The major obstacle to the success of chemotherapy in cancer treatment is the development of tumor drug resistance. In addition, chemotherapy is not specific and the dose of the drug that reaches the tumor may be as little as 5%–10% of the total dose as it accumulates in normal organs [1].

Cancer gene therapy is a promising approach for cancer treatment. Gene therapy was initially conceived as a treatment for inherited diseases, but today up to 70% of clinical trials of gene therapy are designed to treat cancer. Gene therapy uses carriers called vectors to deliver the therapeutic gene to the patient's target cells. Currently, the most common vectors are eukaryotic viruses because they can enter cells as part of the natural infection process. Eukaryotic viruses are, unquestionably, superior vectors for gene transfer, but have had limited success in systemic gene therapy as they are taken up by the liver and reticulo-endothelial system, they have broad tropism for healthy tissues and they may lose efficacy due to the presence of neutralising antibodies [2]. A local vector delivery through intra-tumoral injection can be used to show proof of efficacy, but in practice clinical benefit for cancer treatment can only be achieved following systemic administration.

In 2006, we were the first to describe a novel systemic gene therapy vector based on bacteriophage (phage) that showed successful targeting of gene delivery to tumors in preclinical models of cancer following intravenous administration. We named this vector adeno-associated virus/phage or AAV/phage (AAVP) [3]. One advantage of using phage vectors over animal viral vectors is that bacteriophage has a safety history profile as it was given to humans to treat bacterial infections, and was approved in 2006 by the US Food and Drug Administration for use as safe antibacterial food additive [4]. Other advantages of phage vectors are their large cloning capacity as well as their simple and economical large-scale production and purification [5]. Finally, phage vectors are easily targetable through tissue specific ligands displayed on their capsid, without the need to ablate any native mammalian tropism. Phage display technology has largely shown that ligands displayed on the phage coat proteins remain intact and preserve their receptor binding properties [6]. Bacteriophage can infect and express genes in bacteria only. Thus the mammalian transgene cassette flanked by full length inverted terminal repeats (ITRs) from the recombinant adeno-associated virus (rAAV), which is mammalian single-stranded DNA virus, was genetically incorporated into the phage single-stranded genome [7]. This hybrid vector (AAVP) has no AAV capsid but phage capsid served to package the hybrid single-stranded genome. As phage has no tropism for mammalian cells, the gene encoding the pIII minor coat protein of the phage was genetically manipulated to display, as a fusion, the cyclic CDCRGDCFC (RGD4C) ligand that allows targeting of the phage capsid to tumors through binding to its α_v_ integrin receptors which are specifically expressed on tumors but barely detectable on the healthy tissues. Consequently, the tumor-targeted RGD4C-AAVP achieved specific gene expression within tumors after intravenous administration resulting in strong antitumor effects in rodents, without harming the healthy tissues [3, 7, 8]. Remarkably, a study carried out by the National Cancer Institute, NCI-USA, with targeted RGD4C-AAVP-TNFα carrying the gene for the cytokine tumor necrosis factor alpha (TNFα), showed specific delivery of TNFα in natural tumors in pet dogs, resulting in eradication of aggressive and sizeable tumors such as fibrosarcoma in a few cases [9]. In brief, RGD4C-AAVP is a novel and promising systemic vector for cancer treatment.

However, while some preclinical studies showed remarkable outcomes, vector performance was also poor in some cases. One reason could be associated with poor intracellular trafficking of the vector as RGD4C-AAVP is still a bacteria virus that has not evolved to deliver genes to mammalian cells. We have previously investigated the intracellular trafficking of RGD4C-AAVP in cancer cells and uncovered barriers to gene delivery by RGD4C-AAVP, such as weak phage attachment to the cell surface [10], poor endosomal escape [11], proteasome degradation [12] and nuclear transport (unpublished data). Proteasome inhibiting drugs and endosmolytic agents could be used to assist the phage to overcome intracellular barriers and subsequently enhance gene delivery. However, these drugs are not usually clinically applicable and have to be used at toxic pharmacological doses in order to be efficient. Anticancer treatment strategies such as conventional chemotherapy have been used to enhance gene therapy [13] and can be tested in combination with RGD4C-AAVP. However, chemotherapy is an invasive and toxic treatment with ferocious chemistry unable to differentiate between cancerous and healthy cells [1]. Herein, we postulated that the natural non-toxic dietary plant product, genistein, with proven anticancer activity can be used to enhance the RGD4C-AAVP-mediated tumor cell killing while preserving its safety attribute. Genistein is an isoflavone present in soy [14], that inhibits the growth and development of several malignancies [15–17]. Epidemiological studies have shown that a soy-rich diet is associated with low risk of breast and prostate cancer [18]. Genistein has already been used in clinical trials to investigate combined therapy of genistein and gemcitabine for the treatment of breast cancer patients and to evaluate genistein together with gemcitabine and erlotinib for the treatment of pancreatic cancer patients [19, 20]. Genistein has similar structure to 17β-estradiol presenting weak estrogenic activity and can compete with 17β-estradiol for the estrogen receptor (ER), thus contributing to the treatment of hormone-related cancers [18]. Other mechanisms mediating the anticancer effect of genistein include the inhibition of mitogen activated protein kinase (MAPK) activation resulting in sensitizing human hepatocellular carcinoma Hep3B cells to TNF-related apoptosis inducing ligand TRAIL-mediated apoptosis [21] and also induction of apoptosis in primary gastric cells by downregulating the antiapoptotic protein B cell lymphoma2 (Bcl-2) and upregulating the proapoptotic Bcl-2 associated X protein (Bax) [22]. Genistein also targets caspases, extracellular signal-regulated kinases 1/2 (ERK1/2), nuclear transcription factor κB (NF-κB), Wingless and integration 1 β-catenin (Wnt/β-catenin), phosphoinositide 3 kinase/Akt (PI3K/Akt) and epidermal growth factor receptor protein tyrosine kinase (EGFR-PTK) signalling pathways resulting in anticancer and therapeutic effects [15].

Furthermore, genistein was shown to increase AAV2-mediated transduction efficiency [23, 24]. Importantly, genistein can interfere with cellular pathways and, as a result, can be used to enhance the gene transfer efficacy of RGD4C-AAVP. For instance, the ability of genistein to inhibit the chymotrypsin-like activity of proteasome [25], can be used to prevent AAVP degradation by the proteasome. Moreover, the ability of genistein to induce G2/M cell cycle arrest could result in increased nuclear transport of gene therapy vectors [26–28]. Consequently, taking into account the safety of genistein, its anticancer activity and interference with cellular pathways, we hypothesized that combination of genistein with our tumor-targeted RGD4C-AAVP biotherapeutic particle would lead to enhanced tumor cell killing along with reduced toxicity. We also examined the effects of genistein on targeted gene transfer by RGD4C-AAVP *in vitro* and in tumor spheroid models, and investigated the mechanism of genistein's effects on RGD4AC-AAVP.

## RESULTS

### Genistein drug treatment boosts cancer cell death by phage-mediated suicide gene killing

**Figure 1:**
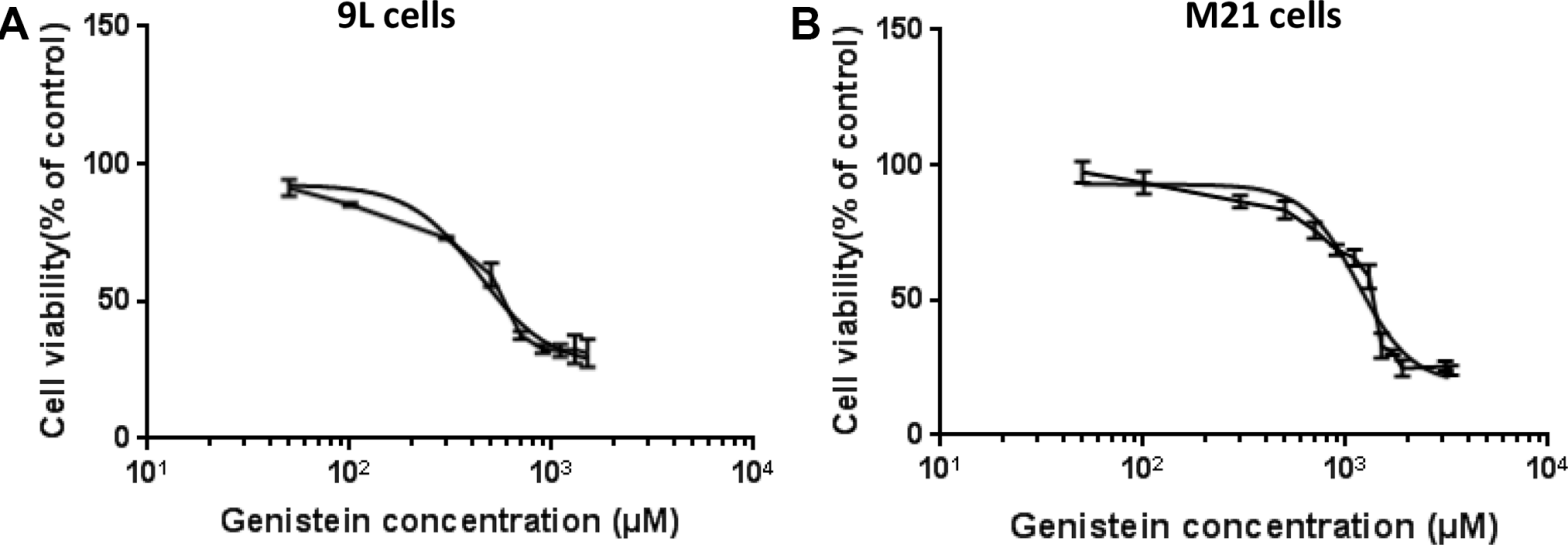
Cytotoxicity of genistein on 9L and M21 tumor cells 9L (**A**) and M21 (**B**) cells were cultured in 96-well plates, then treated with increasing concentrations of genistein ranging from 50 to 3300 μM for 2 hours. Next, cells were grown for further 48 hours without the drug. Cell survival was determined by using the MTT assay and expressed as percentage of cells counted in parallel cultures without the drug. The IC_50_ dose of genistein determined by GraphPad Prism using nonlinear regression was 438.5 μM for 9L cells and 1148 μM for M21 cells. The X-axis is in the log(10) scale and the data fitted to Hill equation. The assay was repeated twice in triplicate and the results shown are representative of one experiment.

First, we sought to assess the cytotoxicity of genistein *in vitro* on 9L rat glioblastoma and M21 human melanoma cell lines. These tumor cells were treated with increasing concentrations of genistein ranging from 50 to 3300 μM for 2 hours and compared to non-treated cells. Subsequently, cell survival was assessed at 48 hours post drug treatment. The data show that tumor cell death raised as the concentration of the drug increased (Figure 1) in both 9L and M21 cancer cells with a more pronounced effect on the 9L glioblastoma cells than M21 melanoma cells. Cytotoxic doses expressed as IC_50_ values, showing the inhibitory concentrations required to induce the cell death by 50%, are shown in Table 1. We found that 50% of cell death in the presence of genistein was induced by ~438.5 μM in 9Lcells (Table 1), while in M21 cells, 50% of cell death was achieved at a dose of over 1148 μM (Table 1). Next, to assess the effect on tumor cell killing by RGD4C-AAVP, we selected genistein concentration of 150 μM for both 9L and M21 cancer cells, as this dose is below the IC_50_, causes little toxicity and was previously reported to enhance gene delivery by eukaryotic viral vectors [24].

**Table 1:** IC_50_ of genistein, curcumin, EGCG, bortezomib and carfilzomib in 9L and M21 cells

	9L cells					M21 cells				
Cytotoxic agent	Genistein	Curcumin	EGCG	Bortezomib	Carfilzomib	Genistein	Curcumin	EGCG	Bortezomib	Carfilzomib
IC_50_ (μM)	438.5	35.05	203.7	1.55	1.034	1148	42.38	361.9	1	1.141

**Figure 2:**
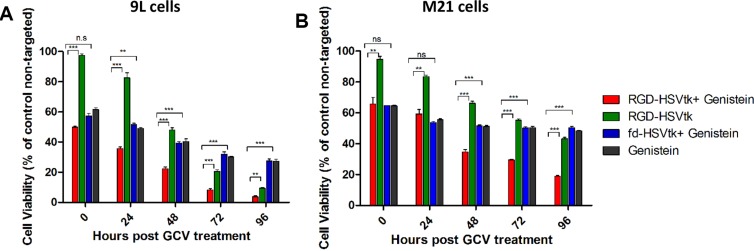
Genistein increased cell death of 9L and M21 tumor cells after transduction with RGD-HSVtk followed by GCV treatment 9L (**Α**) and M21 cells (**B**) grown in 48 well-plates (60–80% confluent) were transduced with RGD-HSVtk targeted vector or control non-targeted fd-HSVtk vector with or without 2 hours pretreatment with genisteιn (150 μΜ). The cells were treated with GCV (20 μM) at day 3 post vector transduction and renewed daily. Cancer cell killing was quantified at 0, 24, 48, 72, 96 hours post GCV treatment. Cells were counted by using the trypan blue exclusion methodology. Results are normalized to control non-targeted fd-HSVtk vector. The experiment was repeated twice in triplicate and the results shown are representative of one experiment.

To test tumor cell killing efficacy, we used the RGD4C-AAVP vector (RGD*-HSVtk)* encoding the gene for the herpes simplex virus type I thymidine kinase (*HSVtk*) mutant SR39 [29] which kills cells in the presence of ganciclovir, GCV (Figure 2). 9L and M21 cells were transduced with RGD-HSVtk or control non-targeted vector fd-HSVtk (without RGD4C) carrying the *HSVtk* gene with or without 2 hours pretreatment with genistein. The cells were then treated with GCV (20 μM) at day 3 post vector transduction. Cancer cell killing was quantified at 0, 24, 48, 72, 96 hours post GCV treatment. Results were normalized to non-targeted vector which did not show any tumor cell death (data not shown). In both cancer cell lines, the combination treatment with genistein and RGD-HSVtk therapy resulted in greater cell killing compared to cells treated with RGD-HSVtk or genistein drug alone (Figure 2). For instance, at 72 hrs post GCV treatment, combination treatment induced 91.6% and 70.5% killing of 9L and M21 cancer cells, respectively (Figure 2), compared to 79.5% and 44.7% death induced by RGD-HSVtk vector alone in 9L and M21 cells, respectively, and 69.8% death and 49.6% death induced by genistein alone in 9L and M21 cells, respectively. These data show that drug treatment of cancer cells with an isoflavone is a promising approach to enhance targeted gene therapy by RGD4C-AAVP.

### Genistein increases targeted reporter gene transfer by the RGD4C-AAVP in 9L and M21 cancer cells *in vitro*

**Figure 3:**
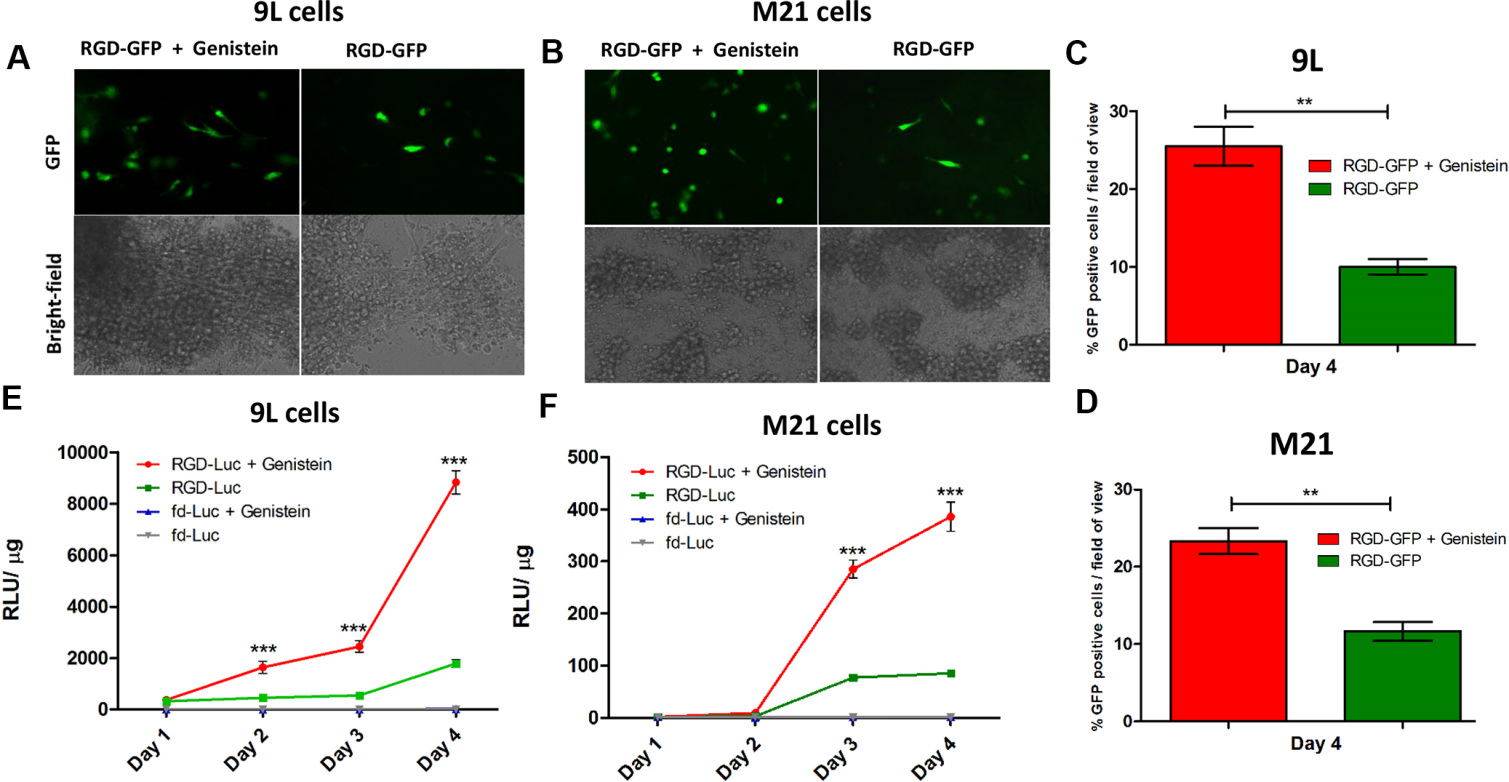
Genistein increased efficiency of gene transfer by AAVP in 9L and M21 tumor cells 9L (**A**) and M21 cells (**B**) were plated on 48-well plates (70–80% confluent) and transduced with RGD-GFP targeted or control non-targeted vectors in serum free medium for 4 hours with or without 2 hour pretreatment with genistein (150 μΜ). GFP expression was monitored by fluorescent microscopy on day 4 post vector transduction, and also evaluated as average of GFP positive cells in five fields of view of 9L (**C**) and M21 (**D**) treated cells. 9L (**E**) and M21 cells (**F**) were plated on 48-well plates (70–80% confluent) and transduced with targeted RGD-Luc or fd-Luc control non-targeted vector in serum free medium for 4 hours with or without 2 hours pretreatment with genistein (150 μΜ). Luciferase measurement assays were performed at day 1–4 post vector transduction and normalized to protein concentration as determined by the Bradford assay. Results are shown as RLU (Relative Luciferase Units) per 1 μg of protein and represent the average from triplicate wells. The experiment was repeated twice in triplicate and the results shown are representative of one experiment.

To gain insight into the improved tumor cell killing by RGD-HSVtk following combination with genistein, we investigated the effect of genistein on gene delivery by RGD4C-AAVP. We first conducted qualitative analyses of transgene expression by using vectors carrying the reporter gene of the *green fluorescent protein* (RGD-GFP) and combined with 2 hours pretreatment with 150 μΜ of genistein (Figure 3). Fluorescent microscopic analysis of GFP expression at day 4 post vector transduction showed that combination treatment with RGD-GFP and genistein resulted in significantly higher GFP expression, compared to RGD-GFP vector alone in both 9L and M21 tumor cells (Figure 3A–3D). Next, to confirm the increased gene delivery by RGD4C-AAVP in combination with genistein, we carried out a quantitative analysis of transgene expression over a time course of 4 days post vector transduction by using RGD4C-AAVP vectors expressing the *firefly luciferase* reporter gene, RGD-*Luc,* (Figure 3E and 3F). Consistently with GFP reporter transgene expression experiments, we observed a significant increase in *luciferase* expression by RGD-Luc vector at various time points post vector transduction by genistein treatment in both 9L and M21 cancer cells compared to cells treated with the vector alone. For instance at day 4 post transduction, combination treatment (RGD-Luc + Genistein) resulted in ~ 4.7 fold and ~3.8 fold increase in luciferase expression in 9L and M21 cells, respectively, compared to RGD-Luc treatment alone. Moreover, in 9L cells, initiation of the luciferase expression occurred as early as day 2 post vector transduction, in the presence of genistein (Figure 3E). Importantly, no luciferase expression was detected in cells transduced with non-targeted fd-Luc vector alone or in combination with genistein, which shows that genistein does not affect the specificity and targeting of the RGD4C-AAVP vector.

Finally, we sought to investigate whether this enhanced AAVP gene delivery by genistein could also occur with other dietary phytochemicals. In this study we selected the phytochemicals curcumin and EGCG (epigallocatechin-3-gallate) known for their anticancer properties. First, we assessed the cytotoxicity of curcumin and EGCG on both 9L and M21 cells and determined the IC_50_ (Supplementary Figures 1 and 2, Table 1). Next, we used non-toxic doses of curcumin and EGCG below the IC_50_ (Table 1), and evaluated 9L and M21 cell transduction by RGD-Luc in combination with curcumin and EGCG. We found that curcumin increased the transduction efficiency of the targeted RGD-Luc in 9L cells, in a dose dependent manner (Supplementary Figure 1C), but not in the M21 cells (Supplementary Figure 1D). Importantly, no effect of curcumin was observed on the control non-targeted fd-Luc vector proving that curcumin did not affect the specificity of the targeted RGD-Luc vector. Finally treatment with EGCG did not increase the transduction efficiency of the RGD-Luc vector in both cell lines (Supplementary Figure 2).

### Evaluation of vector cellular entry following genistein pretreatment

**Figure 4:**
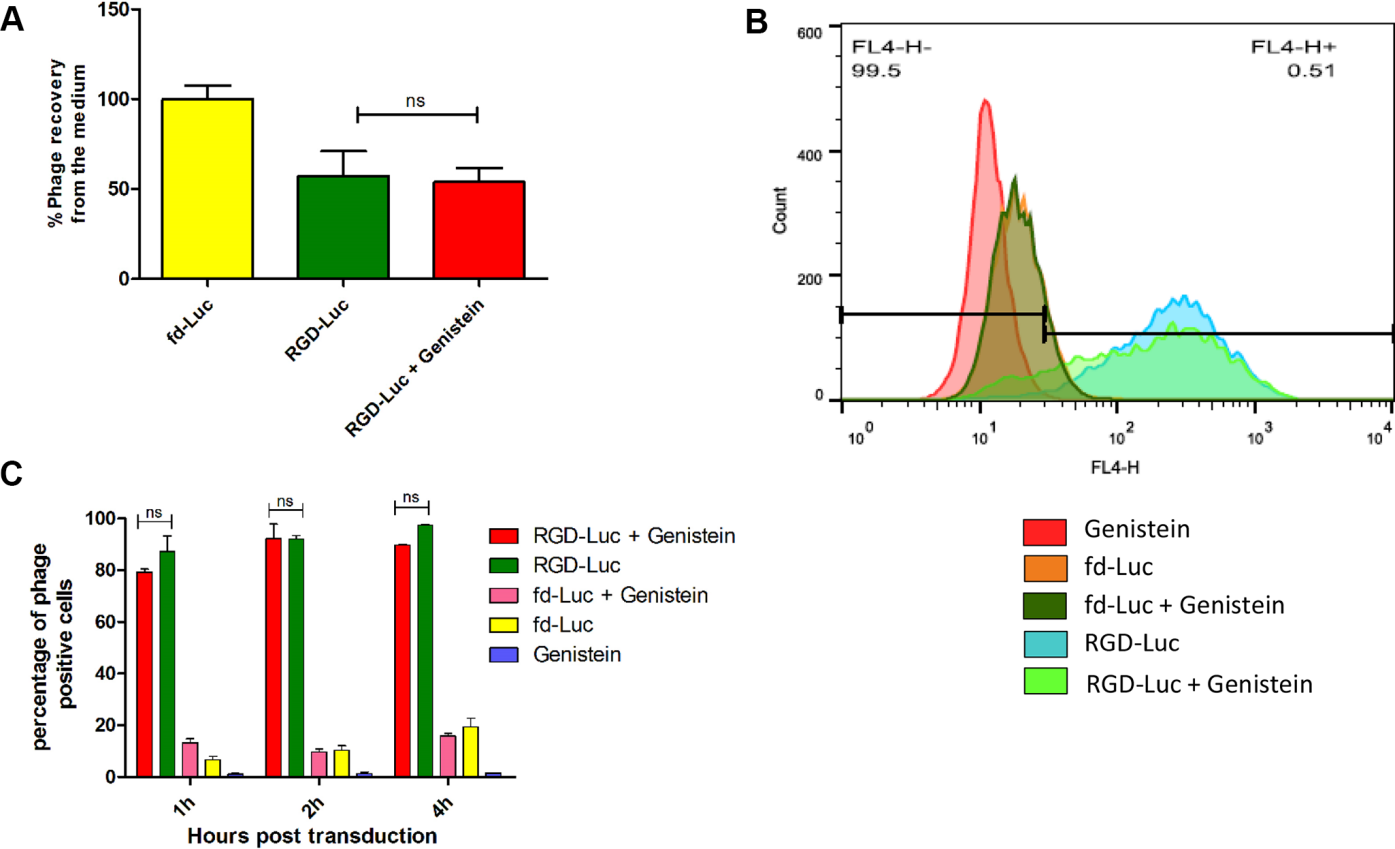
Analysis of cellular attachment and internalization of AAVP vector in combination with genistein (**A**) Cellular attachment of RGD4C-GFP was evaluated by titrating the unbound phage in the supernatant of 9L cells following bacterial colony counting. The experiment was repeated twice in triplicate and the results shown are representative of one experiment. (**B**) Evaluation of vector internalization in the presence or absence of genistein. Graph showing FACS results after immunostaining of 9L cells treated with vectors in the presence or absence of genistein. (**C**) Graph showing the percentage of positive cells according to FACS data at three different time points (1, 2 and 4 hours) post incubation with vector. The experiment was carried out in triplicate.

After demonstrating that the increased tumor cell killing by RGD4C-AAVP observed in combination with genistein was associated with enhanced RGD4C-AAVP-mediated gene expression, we set out to gain further understanding into the mechanism of this enhanced gene transfer. Therefore, we sought to investigate the effect of genistein on steps involved in gene transfer. It is well established that vector-mediated gene delivery depends on several steps where the vector needs to access the cell surface to bind to its receptor, followed by cell internalization and intracellular trafficking, then transport to the nucleus for gene expression to occur [30, 31]. We first examined the effect of genistein on vector attachment to the surface of cells, as we previously reported that gene transfer by RGD4C-AAVP is hindered by its weak accessibility to the surface of tumor cells [10]. Hence, we quantified the free cell-unbound phage in the supernatant above the adherent cells by infection of host bacteria followed by colony counting (Figure 4A). An amount of 52%, of input phage particles, was recovered from the supernatant of cells treated with the RGD-GFP vector showing that a fraction of 48% of input phage was bound to the surface of tumor cells. However, pretreatment with genistein had no effect on vector attachment to the cell surface (Figure 4A). The non-targeted fd-GFP vector showed no attachment to the surface of tumor cells with 100% recovery. Finally, internalization assays revealed that combination of vector with genistein did not increase entry of the RGD4C-AAVP into cancer cells (Figure 4B and 4C). These data show that genistein has no effect on cell attachment and internalization of the RGD4C-AAVP viral particles.

### Genistein protects RGD4C-AAVP from proteasome degradation

**Figure 5:**
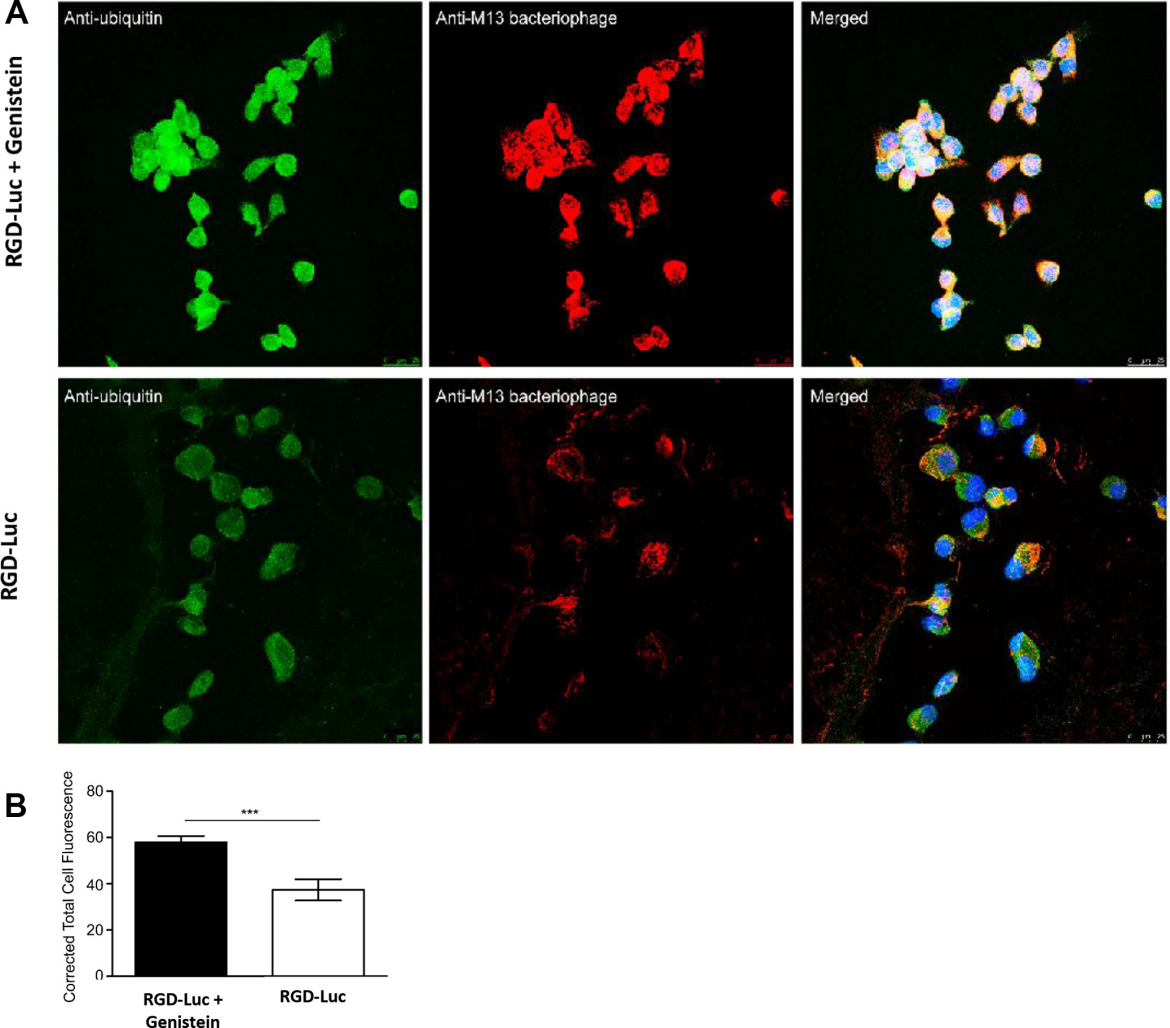
Polyubiquitination of RGD4C-AAVP particle is increased by genistein (**A**) 9L cells were transduced by targeted AAVP (RGD-Luc) in serum-free medium for 4 hours with or without pretreatment with genistein (150 μM). 6 hours post transduction, RGD-Luc was detected using rabbit anti-M13-phage primary and goat anti-rabbit AlexaFluor-647 secondary antibodies (shown in red) and ubiquitin was stained using mouse anti-ubiquitin primary and AlexaFluor-488 secondary antibodies (shown in green). Samples were analyzed by confocal microscopy and representative sections are shown. (**B**) Corrective total cell fluorescence analysis of ubiquitination in single optical sections using ImageJ. Data represent the mean ± standard error of the mean (s.e.m.) of five independent optical sections.

After ruling out the effect of genistein on vector cell entry, we sought to determine whether genistein improves intracellular persistence of the RGD4C-AAVP. We investigated the effect of genistein pretreatment of tumor cells on vector protection against proteasome degradation as we previously reported that proteasome is a barrier to gene transfer by RGD4C-AAVP vectors [12], and because genistein was found to possess proteasome-inhibitory activity [25]. 26S proteasome targets the degradation of polyubiquitinated protein substrates; thus inhibition of proteasome degradation by genistein would lead to accumulation of AAVP ubiquitination [32]. Therefore, we investigated whether genistein increases polyubiquitination of AAVP phage coat proteins. 9L tumor cells were transduced with RGD-Luc vector alone or following pretreatment with genistein. Next, the cells were analyzed for co-localization of AAVP coat proteins and ubiquitin by immunofluorescence as reported [33], by using antibodies against ubiquitin and phage coat proteins (Figure 5). Confocal microscopic analysis showed strong co-localization of ubiquitin and AAVP coat proteins in cells treated with combination of vector and genistein (Figure 5). These data demonstrate that combination treatment results in accumulation of polyubiquitinated AAVP particles compared to treatment with the targeted vector alone, indicating that genistein can increase the gene transfer efficiency by inhibiting proteasome-mediated degradation of AAVP particles.

### Genistein enhances nuclear localisation of the RGD4C-AAVP vector genome

**Figure 6:**
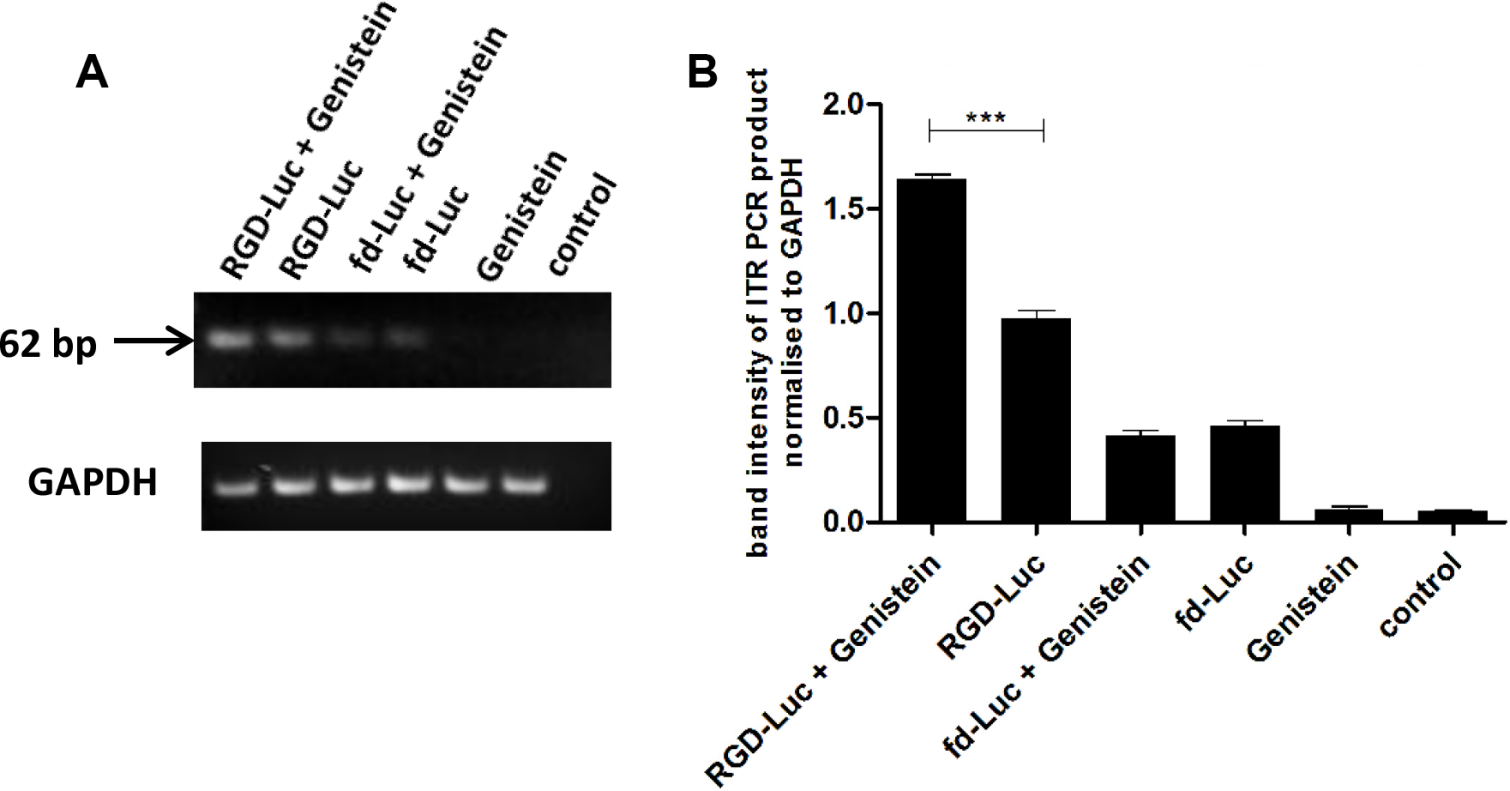
Genistein enhances AAVP vector genome accumulation in the nucleus (**A**) 9L cells were plated on 48-well plates (70–80% confluent) and transduced with targeted RGD-Luc or fd-Luc control non-targeted vector in serum free medium for 4 hours with or without 2 hours pretreatment with genistein (150 μΜ). On day 4 after transduction, cells were harvested and nuclei extracted. Subsequently, DNA was extracted and used as template (10 ng of DNA) for PCR of the ITR domain in order to semi-quantify the amount of vector in the nucleus with or without pretreatment with genistein. Similar amount of DNA (10 ng) was used as template for PCR of the GAPDH gene. The experiment was carried out in triplicate. (**B**) The band intensity of the ITR PCR product was quantified using ImageJ software and normalized to GAPDH. PCR of the ITR domain was repeated three times and shown is the average.

Finally, we examined vector's genome accumulation in the nucleus to check whether enhanced resistance of vector to proteasome degradation would result in enhanced nuclear localisation of vector's genome. Additionally, genistein has been reported to induce G2/M cell cycle arrest, which results in pronounced opening of the nuclear pores allowing better nuclear transport of gene delivery vectors [26–28]. Thus, we evaluated the nuclear accumulation of the AAV2 transgene cassette, since gene expression by RGD4C-AAVP is mediated through its AAV2 transgene cassette. 9L cells were transduced with RGD-Luc or fd-Luc non-targeted vector with or without 2 hours pretreatment with genistein (150 μΜ), and harvested at day 4 post transduction. Next, the nuclei were extracted from cells, followed by PCR using primers reading within the AAV2 ITR domain, as previously described [34], in order to semi-quantify the amount of vector genome in the nucleus (Figure 6). The data revealed a PCR product at the expected size, and electrophoresis gel analysis showed increased intensity of the ITR-derived PCR product when RGD-Luc vector was used in combination with genistein (Figure 6A). Then product quantification of the band intensities using ImageJ software confirmed that combination of genistein with the targeted vector resulted in significant increase of vector DNA in the nucleus (Figure 6B).

### Evaluation of efficacy of genistein and RGD4C-AAVP combination in a three-dimensional (3D) multicellular tumor spheroid

**Figure 7:**
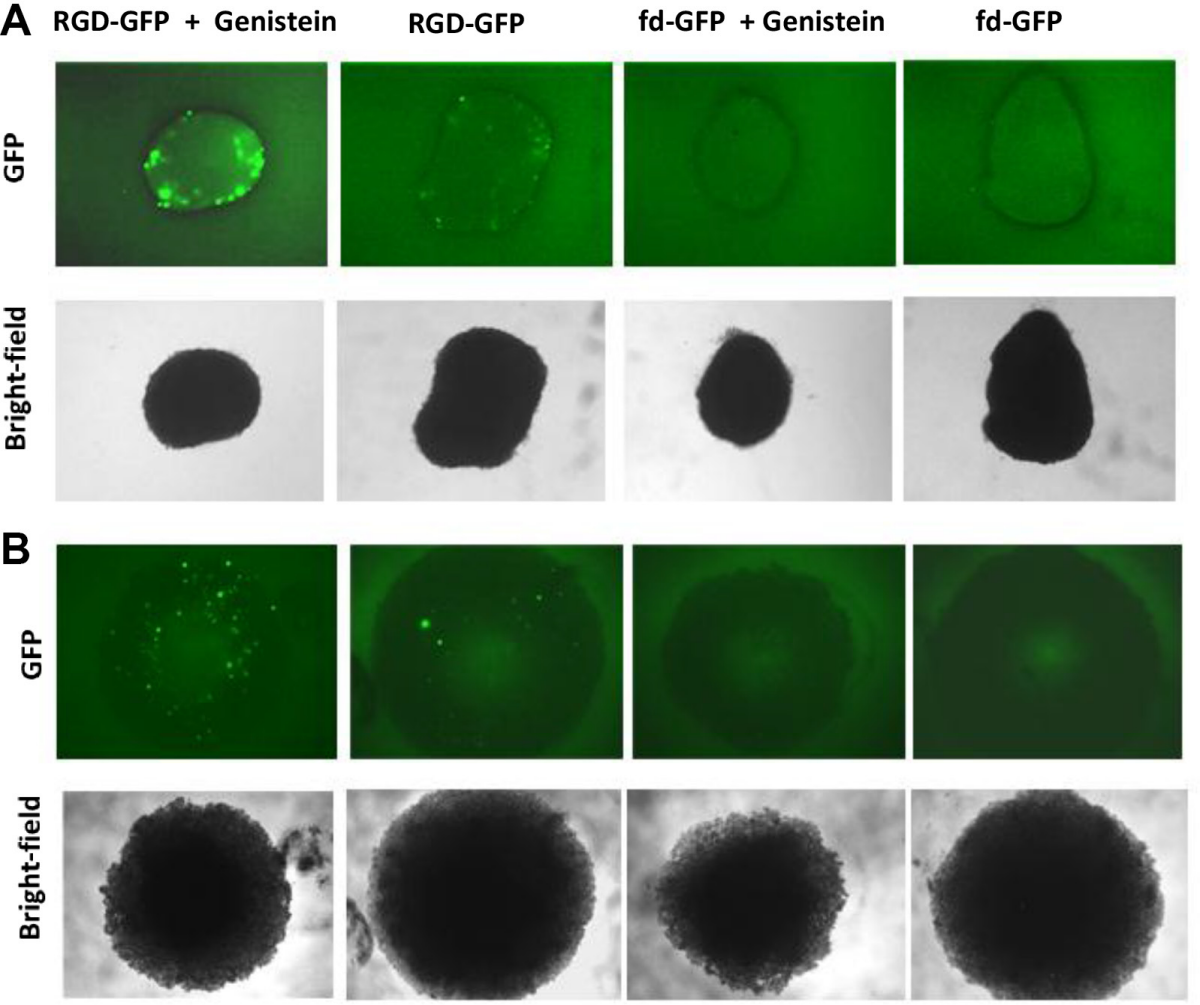
Genistein increased RGD4C-AAVP-mediated gene transfer in 9L and M21 tumor spheroids 9L (**A**) and M21 (**B**) cells (5 × 10^3^) were seeded into a 96-well ultra-low attachment surface plate in 200 μL complete medium. After 48 hours of incubation, a spheroid was formed in each well. Spheroids were then transduced with RGD-GFP or control fd-GFP non-targeted vector with or without pretreatment with genistein (150 μΜ). GFP expression was evaluated with fluorescent microscopy at day 10 post vector transduction.

After showing that genistein dramatically increased RGD4C-AAVP mediated targeted killing of tumor cells *in vitro*, we set up to assess the efficacy of this combination in 3D tumor spheroids that simulate the 3D tumors more accurately. The 3D tumor spheroids are considered valid models to recapitulate features of solid tumors and were used in this study to evaluate and confirm the efficacy of gene therapy by the targeted RGD4C-AAVP in combination with genistein. Since the increased tumor cell killing *in vitro* of the combination genistein and RGD4C-AAVP was associated with the enhancing effect of genistein on RGD4C-AAVP-mediated gene transfer, we first assessed the efficacy of gene transfer using vector carrying the *GFP* reporter gene to allow microscopic imaging of GFP expression within the 3D models of 9L and M21 tumor spheroids (Figure 7). The 9L and M21 tumor spheroids were transduced with targeted RGD-GFP or control non-targeted fd-GFP with or without pretreatment with genistein (150 μΜ). GFP expression was monitored with fluorescent microscopy over a period of 10 days to allow detectable gene expression by the RGD-GFP in the spheroids. While the targeted RGD-GFP showed minimal GFP expression in the spheroids at day 10 post transduction, combination treatment (RGD-GFP + Genistein) yielded dramatic increase in GFP expression compared to RGD-GFP treatment alone in both 9L and M21 spheroids (Figure 7).

**Figure 8:**
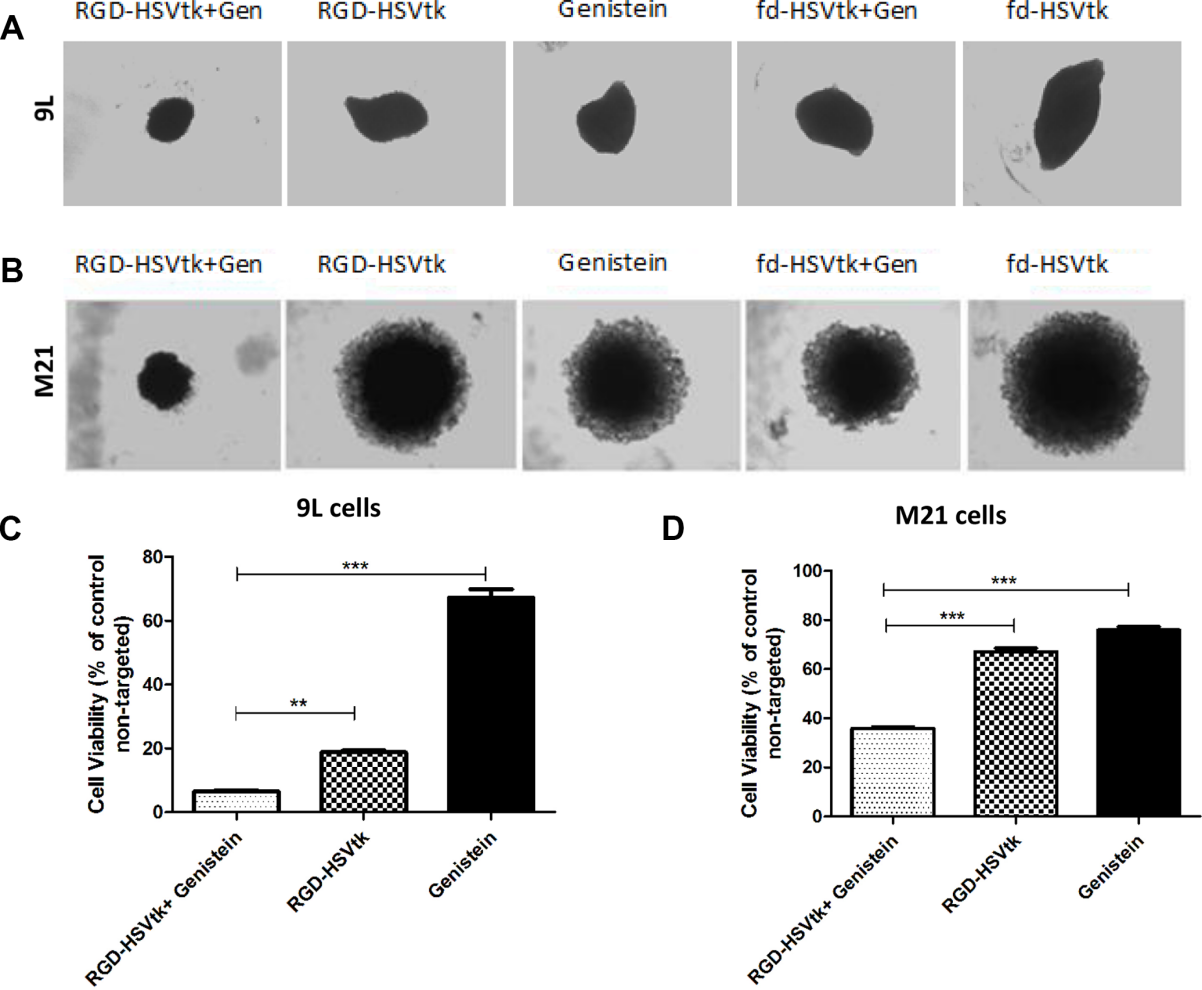
Antitumor efficacy of RGD4C-AAVP in combination with genistein in 3D tumor spheroid models Bright-field images showing the size of 9L (**Α**) and M21 (**B**) spheroids following transduction with RGD-HSVtk or fd-HSVtk non-targeted vector with or without pretreatment with genistein (150 μΜ). GCV was added to the spheroids at day 5 post vector transduction and renewed every 2 days. Images were taken at day 5 or day 7 post GCV treatment for 9L and M21 spheroids, respectively. (**C**) Evaluation of cell viability in 9L spheroids at day 5 post GCV treatment. (**D**) Evaluation of cell viability in M21 spheroids at day 7 post GCV treatment. Cell viability was assessed by using the Cell Titer-Glo cell viability assay. The experiments were repeated twice in triplicate and the results shown are representative of one experiment.

Next, application of *HSVtk*/GCV suicide gene therapy on rat 9L and human M21 tumors *in vitro* resulted in regression of the 9L and M21 spheroid volumes by combination of genistein with the targeted RGD-HSVtk upon GCV treatment, compared to individual treatments with RGD-HSVtk or genistein alone (Figure 8A and 8B). Subsequently, measurement of cell viability in 9L spheroids showed that combination of genistein plus RGD-HSVtk achieved higher tumor cell killing ~93%, than the targeted RGD-HSVtk or genistein alone that induced ~81% and ~33% cancer cell killing, respectively (Figure 8C). In M21 spheroids, measurement of cell viability showed that combination of genistein plus RGD4C-HSVtk achieved higher tumor cell killing ~65% than the targeted RGD-HSVtk or genistein alone that induced ~33% and ~24% cancer cell killing, respectively (Figure 8D). These findings clearly establish that combination of genistein with RGD4C-AAVP-mediated gene therapy greatly increases its potential as a gene therapy vector.

## DISCUSSION

We have demonstrated that genistein pretreatment of tumor cells from different histopathological types and species resulted in enhanced targeted tumor cell killing by RGD4C-AAVP-mediated *HSVtk* and GCV suicide gene therapy in 2D tissue culture and 3D tumor spheroid settings. Then, we found that treatment with genistein of 9L and M21 cancer cells increased *GFP* and *Luc* reporter gene expression, which demonstrates that the enhanced tumor cell killing of RGD4C-AAVP by genistein is associated with increased *HSVtk* gene expression. Moreover, we have investigated the mechanisms linked with this increased reporter gene expression and tumor cell killing. Importantly gene transfer by RGD4C-AAVP remains targeted in the presence of genistein, indicating that the tumor specificity of RGD4C-AAVP is not affected by genistein. We also found that genistein increases polyubiquitination of AAVP particles and accumulation of vector genome in the nucleus. These data suggest that genistein may bestow an advantage in gene expression from RGD4C-AAVP by means of increased vector accumulation in the nucleus and vector protection from proteasome degradation, or perhaps a combination of these two non-mutually exclusive mechanisms.

Our findings of increased RGD4C-AAVP-mediated cancer gene therapy are in agreement with a previous report showing enhanced cancer cell killing by a mutant oncolytic adenovirus in combination with genistein [35]. The observed difference in cell viability between 9L and M21 cell lines can be attributed partially to the fact that 9L cells can get transduced more easily compared to M21 cells and also to the bystander effect of the HSVtk/GCV in 9L cells [36]. The enhanced RGD4C-AAVP-mediated *GFP* and *Luc* gene expression by genistein is also consistent with previous reports that genistein increased AAV2-mediated gene transfer in the human HeLa cervical carcinoma cells [23]. Moreover, the authors reported that genistein enhanced accumulation of the dephosphorylated form of single-stranded D-sequence-binding protein (ssD-BP), which facilitates the conversion of single to double stranded DNA resulting in improved AAV gene expression [23]. Importantly, our findings that genistein increased AAV2 PCR product in the nuclear fraction of cells treated with RGD4C-AAVP are consistent with that study, as gene expression by RGD4C-AAVP is mediated by the AAV2 transgene cassette, incorporated within the phage genome. The increased nuclear accumulation of AAVP genome, upon genistein pre-treatment, provides an additional mechanism that could further explain the improved AAVP-mediated gene expression by genistein. One explanation is that pretreatment with genistein enhances the size of the nuclear pores, as genistein was reported to induce G2/M cell cycle arrest, during which nuclear localisation of gene therapy vectors can be enhanced [26–28].

Moreover, resistance to proteasome degradation upon genistein pretreatment should result in better intracellular persistence and availability of the RGD4C-AAVP particles to be transported to the nucleus. These data also suggest that genistein increases the RGD4C-AAVP-targeted gene expression, at least in part, through inhibition of proteasome-mediated degradation of the RGD4C-AAVP particles. Indeed, genistein was reported to inhibit the chymotrypsin-like activity of proteasome in purified 20S proteasome and 26S proteasome [25]. Also, this is in agreement with our previous report showing that the proteasome inhibitors MG132 and LLnL increased persistence of RGD4C-AAVP particles in cancer cells *in vitro* and in tumors *in vivo* and subsequently improved gene expression from the vector both *in vitro* and *in vivo* [12]. MG132 and LLnL are peptide aldehyde inhibitors that reversibly inhibit the 26S proteasome activity and most widely used in proteasome inhibition studies. The clinically used proteasome inhibitors bortezomib and carfilzomib, in patients with myeloma and other haematological malignancies, were reported to increase transduction efficiency of AAV vectors in hepatic cells and HeLa cell line [37, 38]. However, bortezomib and carfilzomib did not show any enhancing effect on the transduction efficiency of RGD4C-AAVP in 9L and M21 cancer cell lines, although using various doses (Supplementary Figures 3 and 4). Compared to MG132 and LLnL, bortezomib and carfilzomib are stronger proteasome inhibitors even when used at low doses, which could result in increased cancer cell distress and subsequent reduction of RGD4C-AAVP gene expression.

Taking into account that genistein doesn't affect vector attachment on the surface of cancer cells nor its internalisation (Figure 4), our data indicate that genistein affects the intracellular fate of AAVP. It is important to note that other mechanisms of action of genistein might also account for its enhancing effect on transduction efficiency of RGD4C-AAVP. For instance, genistein was reported to modulate the lysosomal metabolism [39]. Given that the endosomal-lysosomal pathway has been identified as an intracellular barrier to efficient transduction by RGD4C-AAVP [11], lysosomal alteration by genistein might facilitate RGD4C-AAVP escape from the lysosomes and subsequently higher nuclear accumulation of AAVP genome and enhanced gene expression.

In conclusion, despite their advantageous natural characteristics, bacteriophage viruses are still considered poor vectors for gene delivery due to their low gene transfer efficiency compared to eukaryotic vectors. The AAVP vector was reported as an improved version of phage-based gene therapy vectors to achieve enhanced gene delivery compared to conventional phage-derived vectors. Although promising, AAVP still has limitations inherent to bacteriophage. In this study we have shown that combining targeted RGD4C-AAVP with genistein significantly improves AVVP-guided gene transfer efficacy and consequently its cancer cell killing as a gene therapy vector. In addition, we elucidated possible mechanisms of increased AAVP-mediated gene expression by genistein. The next preclinical step will be taken to assess efficacy of this combination treatment in tumor-bearing mice. Given that genistein and AAVP have been demonstrated to cross the blood-brain barrier [40, 41], this combination treatment has potential applications for brain tumors. Our study indicates that combination of RGD4C-AAVP and genistein, is a promising strategy that can be considered for future clinical applications of targeted systemic gene therapy with RGD4C-AAVP in cancer patients.

## MATERIALS AND METHODS

### Cells and reagents

The rat 9L glioblastoma cells were a gift from Dr Hrvoje Miletic (University of Bergen, Norway), while the human M21 melanoma cells were from the American Type Culture Collection (ATCC). Both cell lines were maintained in a humidified incubator at 37°C in 5% CO_2_ and cultured in Dulbecco's Modified Eagle's Medium (Sigma) supplemented 10% fetal bovine serum (FBS, Sigma), penicillin (100 units/ml, Sigma), streptomycin (100 μg/ml, Sigma), and L-glutamine (2 mmol/l, Sigma). Genistein was purchased from Sigma and a stock solution (150 mM) was prepared in dimethyl sulfoxide (DMSO, Sigma).

### MTT [3-(4,5-Dimethylthiazol-2-yl)-2,5-Diphenyltetrazolium Bromide] assay

Cytotoxicity of genistein was assessed by using the MTT assay (colorimetric assay to measure cell viability based on mitochondrial activity, Sigma). 9L and M21 cells were plated in 96-well plates at a density of 4 × 10^3^ cells per well. Next day, complete medium (100 μl) containing different concentrations of genistein were added to cells in triplicates. After 2 hours treatment, the genistein-containing medium was removed and replaced with 100 μl fresh medium. MTT assay was carried out after 48 hours.

### Production, purification and titration of AAVP vectors

AAVP vectors were generated as previously reported [7] by inserting the mammalian cassette from AAV2 containing the reporter or therapeutic genes into the fUSE5 plasmid derived from the filamentous fd-tet bacteriophage. AAVP viral particles were produced and purified from the culture supernatant of *Escherichia Coli* K91 host bacteria as previously described [7], then sterile-filtered through 0.45 μM filters. The titration was carried out by preparing serial dilutions of the AAVP and infecting K91 host bacteria, which subsequently form colonies when plated on selective Luria-Bertani agar. The AAVP titre is calculated by counting the number of colonies multiplied by the dilution of the AAVP and is expressed as bacterial transducing units (TU/μl) as previously described [7].

### *In vitro* cell transduction by AAVP vectors

9L and M21 cells were counted, then plated on 48-well plates and grown for 48 hours until they reach 70–80% confluence. Genistein was diluted in complete medium and added to the cells (as indicated) at a final concentration of 150 μM [24]. After 2 hours treatment, genistein-containing medium was removed and cells were washed with serum-free medium. Subsequently, cells were incubated with targeted RGD4C-AAVP or control non-targeted AAVP vectors (10^6^ TU/cell for *HSVtk* carrying vectors, 2.5 × 10^5^ TU/cell for *GFP* carrying vectors, and 10^4^ TU/cell for *Luc* carrying vectors) in serum-free medium (150 μl total volume per well) at 37°C in the CO_2_ incubator for 4 hours and manually rotated every 30 min during incubation. After 4 hours, 350 μl of complete medium were added to make the total volume up to 500 μl per well. Cell transduction efficacy *in vitro* was evaluated by using the green fluorescent protein (*GFP*) and the firefly luciferase (*Luc*) reporter genes. The variation in the TU/cell amounts used between vectors carrying different transgenes is related to the sensitivity of the assay. For instance, given the sensitivity of the luciferase assay, we used 10^4^ TU/cell to avoid saturation of the signal.

### Reporter gene assays

Quantification of luciferase expression was carried out by using the Steady-Glo luciferase assay (Promega). Medium was removed and 110 μl of Glo Lysis buffer, was added per well of 48 well plate. After 10 min incubation, 50 μl of the cell lysate was transferred to a 96-well white opaque microplate (BD, Falcon) and mixed with an equal volume of Steady-Glo^®^ luciferase substrate (Promega). After 10 min the plate was read using a Promega Glomax plate reader. Luciferase expression was normalized to 100 μg protein levels from cell lysates as determined by the Bradford assay (Sigma). Results are shown as Relative Luciferase Units (RLU) per 100 μg of protein. GFP expression was visualized using a Nikon Eclipse TE2000-U fluorescence microscope.

### Determination of tumor cell killing *in vitro*

9L and M21 cells were seeded in 48 well-plates for 48 hours until they reach 70–80% confluence. Then, cells were transduced with RGD4C-AAVP or control non-targeted vector carrying thymidine kinase of the herpes simplex virus type 1 (RGD-HSVtk) gene with or without 2 hours pretreatment with genistein (150 μΜ). Ganciclovir (GCV, Sigma) was added to the cells (20 μM) at day 3 post vector transduction and renewed daily. Cancer cell killing was quantified at 0, 24, 48, 72, 96 hours post GCV treatment. Cells were counted by using the trypan blue exclusion methodology. Results were normalized to non-targeted vector (fd-HSVtk).

### 3D model of multicellular tumor spheroid culture and treatment

9L and M21 multicellular tumor spheroids were prepared by seeding 5 × 10^3^ cells into a 96-well ultra-low attachment surface plate (Corning) in 200 μL complete medium. After 48 hours of incubation, a spheroid was formed in each well. Then, after removing 100 μl of media, spheroids were incubated with targeted vectors or control non-targeted vectors in 100 μl complete medium with or without 2 hours pre-treatment with genistein (150 μΜ). 24 hours later, the medium was replaced with 200 μl complete medium and renewed every 3 days by fresh complete medium. *GFP* gene expression was evaluated using fluorescent microscopy at day 10 post transduction. When spheroids were transduced with vectors carrying the *HSVtk* gene, GCV (20 μΜ) were added on day 5 post vector transduction and renewed every 2–3 days. Cell viability in the spheroids was evaluated post GCV treatment by using CellTiter-Glo assay (Promega). First, medium was removed and then 100 μl of Glo Lysis buffer, 1x, were added in each well. After 30 min incubation, the spheroids were dissolved by pipetting in the lysis buffer or using a sonicator. 50 μl of the lysate was then transferred to a 96-well white opaque microplate (BD Falcon), mixed with an equal volume of CellTiter-Glo substrate (Promega) and read with a Promega Glomax plate reader.

### Nuclei extraction

9L cells were plated on 48-well plates (70–80% confluent) and transduced with RGD4C-AAVP carrying the *Luc* gene (RGD-Luc) or control non-targeted fd-Luc vectors in serum-free medium for 4 hours with or without 2 hours pretreatment with genistein (150 μΜ). On day 4 after transduction, cells were harvested and nuclei were extracted as previously reported [42]. Cells were washed with phosphate buffered saline (PBS, Sigma), trypsinized and then pelleted by centrifugation at 1500 × g for 5 min. The pellet was washed with PBS and pelleted again by centrifugation at 1500 × g for 5 min. Subsequently, the pellet was resuspended in hypotonic buffer (20 mM Hepes-KOH, pH 8.0, 5 mM KCl, 1.5 mM MgCl2, 5 mM Sodiumbutyrate, 0.1 mM dithiothreitol [DTT]) and lysed by dounce homogenization. Nuclei were collected by centrifugation (10 min, 16,000 × g, 4°C) and resuspended in nuclear extraction buffer (15 mM Tris-HCl, pH 7.5, 1 mM EDTA, 0.4 M NaCl, 10% sucrose, 1 mM DTT). DNA was extracted using Phenol/Chloroform/Isoamyl alcohol mixture (Sigma) and precipitated by 100% ethanol (Sigma). The precipitates were washed with 80% ethanol and resuspended in 10 mM Tris-HCl, pH 8.5.

### Semi-quantitative polymerase chain reaction (PCR) analysis

After extraction, DNA was used as a template for PCR targeting the ITR domain of the vector in order to semi-quantify the amount of vector in the nucleus with or without pretreatment with genistein. The same amount of DNA was used as template for PCR of the *GAPDH* gene. PCRs were performed in a 25 μl volume containing 2 mM MgCl_2_, 0.4 mM dNTP mixture (Invitrogen), 0.2 μΜ forward ITR primer (fwd ITR primer, 5′-GGAACCCCTAGTGATGGAGTT-3′), 0.2 μΜ reverse ITR primer (rev ITR primer, 5′-CGGCCTCAGTGAGCGA-3′), 10 ng DNA template and 1 Unit of Q5 polymerase (New England Biolabs). The PCR program contained an initial denaturation step at 98°C for 10 min followed by 35 cycles of denaturation at 98°C for 15 sec, annealing at 61.5°C for 30 sec, and extension at 72°C for 1 min, with a final extension after the last cycle at 72°C for 5 min [34]. The 62-bp PCR product was analyzed on a 4% agarose gel. The band intensity of the PCR product was quantified using ImageJ software and normalized to GAPDH PCR product (Fwd *gapdh* primer: 5′-ATGAATACGGCTACAGCAACAGG-3′, Rev *gapdh* primer: 5′-CTCTTGCTCAGTGTCCTTGCTG-3′).

### Attachment assay

9L and M21 cells were seeded in 48-well plates and grown for 48 hours until they reach 70–80% confluence. Genistein was diluted in complete medium and added to the cells (as indicated) at a final concentration of 150 μM [24]. After 2 hours treatment, genistein-containing medium was removed and cells were treated with targeted or control non-targeted AAVP vectors (10^6^ TU/cell) in serum free medium (150 μl total volume per well). The plates were placed on ice for 1 hour to prevent internalization of AAVP. Next, the supernatants were collected and serially diluted in PBS. The amount of AAVP particles in the supernatant was quantified using the K91 bacterial infection method, as previously described [7].

### Internalization assay

Internalization assay was performed as previously described [10]. After 2 hours treatment with medium containing genistein (150 μΜ), cells were incubated with targeted and control non-targeted vectors carrying the *Luc* gene(10^4^ TU/cell) in serum-free medium for 1, 2 and 4 hours at 37°C. Then, cells were cooled on ice to stop endocytosis and washed 3 times with PBS to remove unbound vectors. Cells were trypsinized for 5 min at 37°C (to remove surface-bound phage) and pelleted by centrifugation at 2000 rpm for 5 min. Subsequently, cells were fixed in 4% paraformaldehyde (PFA, pH 7.2, Sigma) for 10 minutes at room temperature. Untreated cells were used as negative controls. Cells were blocked with 0.1% saponin in 2% bovine serum albumin in PBS (BSA-PBS) for 30 min. To detect internalized phage-derived vectors, cells were stained with a polyclonal rabbit anti-M13-bacteriophage antibody (1:1000, Sigma) in 0.1% saponin in 1% BSA-PBS for 1 hr at room temperature. Cells were washed three times (pelleted and resuspended) in 0.1% saponin in 1% BSA-PBS. Subsequently, cells were incubated with the secondary antibody, goat anti-rabbit AlexaFluor-647 (dilution 1:500, Life Technologies) for 1 hour at room temperature. Finally, cells were washed twice with 0.1% saponin-PBS and resuspended in PBS before analysis.

Fluorescence-activated cell sorting (FACS) analysis was carried out using a FACscalibur Flow cytometer (BD Biosciences). The mean fluorescence intensity was measured for at least 10,000 gated cells per triplicate well. Results were analyzed using Flowjo (TreeStar) software.

### Immunofluorescence staining

9L cells were seeded on 18 mm^2^ coverslips in 12-well plates. After 48 hours, cells at approximately 80% confluent were incubated with RGD-Luc vector (10^6^ TU/cell) in serum-free medium with or without 2 hours pre-treatment with genistein (150 μM). After 4 hours, 350 μl of complete medium were added to make up a total volume of 500 μl per well. After 2 hours (6 hours post transduction), cells were washed with PBS and fixed in PBS containing 4% paraformaldehyde (Sigma). Cells were then incubated in 50 mM Ammonium Chloride for 5 min, permeabilized with 0.2 % Triton X-100 (Sigma), washed, and blocked with PBS containing 2% BSA. Subsequently, cells were incubated with a rabbit anti-M13 bacteriophage (1:1000, Sigma) and mouse anti-ubiquitin (1:200, Invitrogen) diluted in 1% BSA at 4°C overnight. Next day, cells were washed with PBS and incubated for 1 hour at room temperature with 1% BSA containing anti-rabbit and anti-mouse Alexa Fluor-conjugated secondary antibodies (1:750, Invitrogen) and also DAPI (1:2000, Sigma). Cells were washed three times in PBS and twice in distilled water, allowed to air-dry and mounted in Prolong Gold antifade reagent (Invitrogen). Images were acquired with a laser confocal microscope (Leica, Germany).

### Statistical analysis

Statistical analysis was performed using GraphPad Prism software (version 5.0). Data are expressed as mean ± standard error of the mean (s.e.m.). *P* values were generated by one-way ANOVA and Tukey tests. *P* values were considered significant when < 0.05 and denoted as follows: *p < 0.05, **p < 0.01, ***p < 0.001, n.s: non-significant.

## SUPPLEMENTARY MATERIALS FIGURES


