# Supplementary material for: The natural dietary genistein boosts bacteriophage-mediated cancer cell killing by improving phage-targeted tumor cell transduction
Source: Oncotarget. 2016 Jul 18;7(32):52135–49. doi: 10.18632/oncotarget.10662 (PMC5239540; doi:10.18632/oncotarget.10662)
Supplement: Supplementary file 1 [file oncotarget-07-52135-s001.pdf]

The natural dietary genistein boosts bacteriophage-mediated cancer cell killing by improving phage-targeted tumor cell transduction

Supplementary Materials

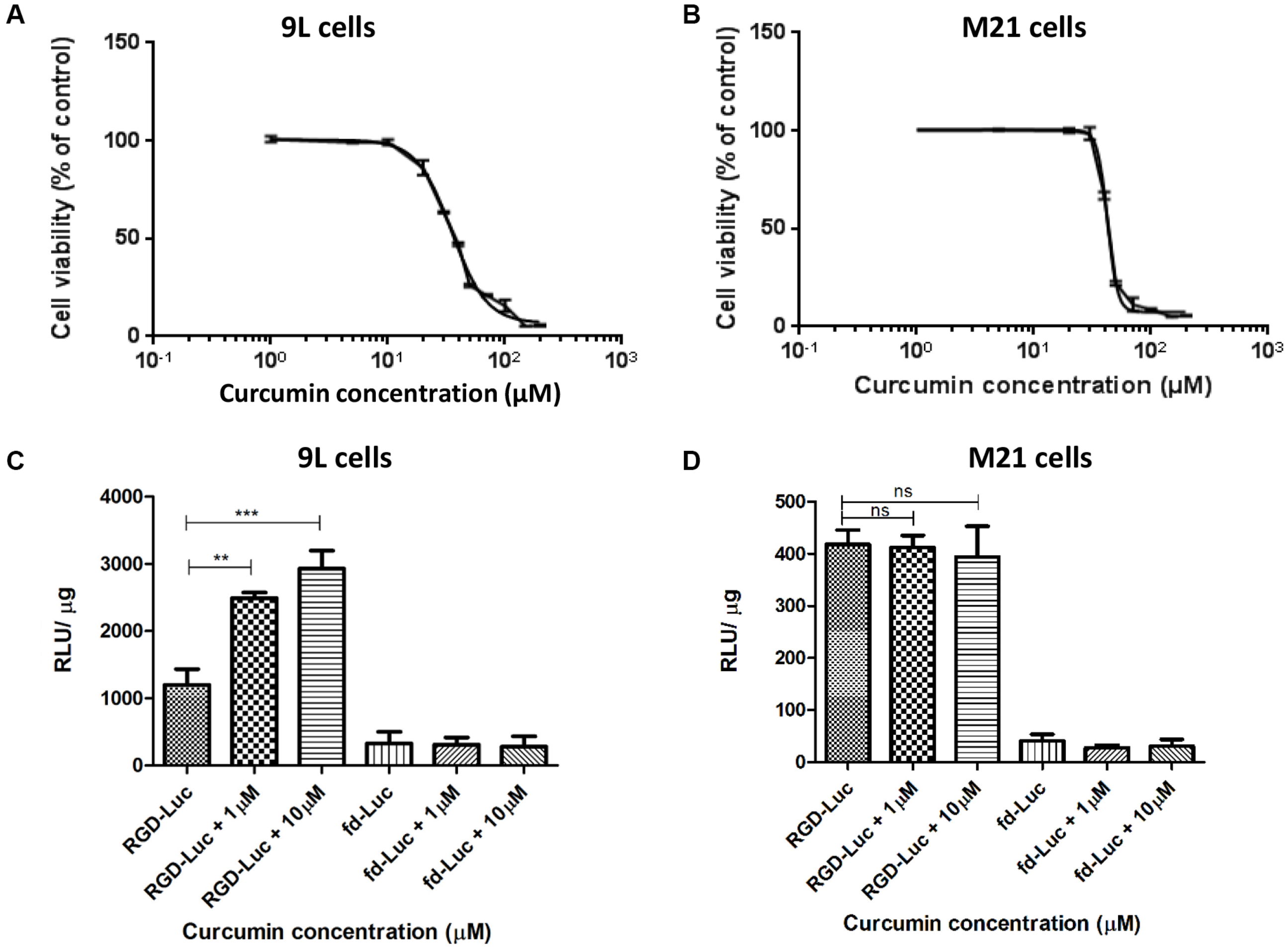

Supplementary Figure S1: (**A, B**) Cytotoxicity of curcumin on 9L and M21 tumor cells. 9L (A) and M21 (B) cells were cultured in 96-well plates, then treated with increasing concentrations of curcumin ranging from 1 to 200 µM for 6 hours. Next, cells were grown for further 48 hours without the drug. Cell survival was determined by using the MTT assay and expressed as percentage of cells counted in parallel cultures without the drug. The IC_{50} dose of curcumin determined by GraphPad Prism using nonlinear regression with variable slope was 35.05 µM for 9L cells and 42.38 µM for M21 cells. The X-axis is in the log(10) scale and the data fitted to Hill equation. The assay was repeated twice in triplicate and the results shown are representative of one experiment. (**C, D**) Determination of optimal curcumin dose in 9L and M21 cells using luciferase assay. 9L (C) and M21 (D) cells were transduced with targeted vector (RGD-Luc) or control non-targeted vector (fd-Luc) in combination with 1 µM or 10 µM of curcumin for 6 hours. Luciferase assay was performed at day 4 post-transduction. The results were normalized to the amount of protein and presented as RLU (Relative Luminescence Units)/µg of protein. Data represent the mean ± standard error of the mean (s.e.m.) of triplicate samples.

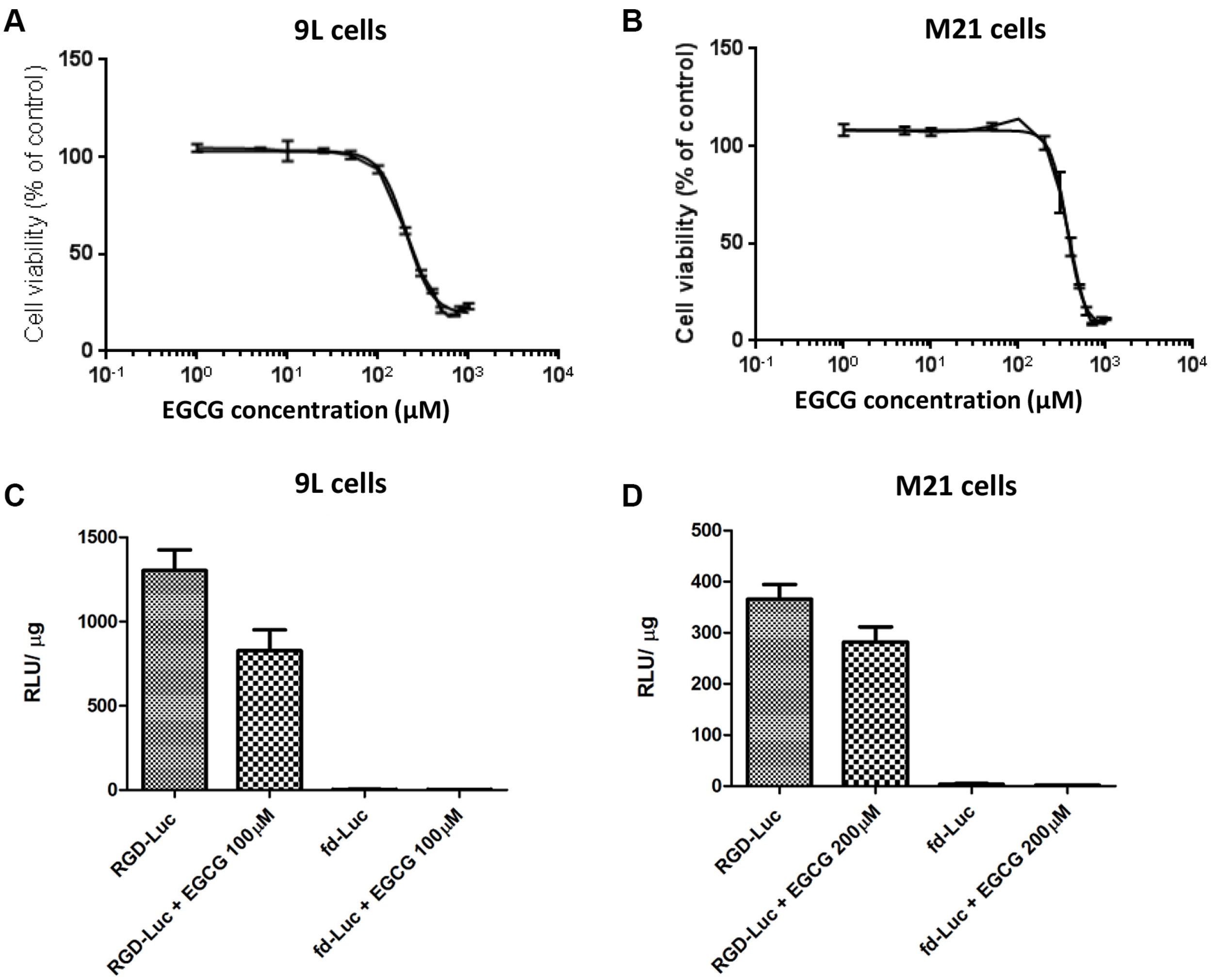

Supplementary Figure S2: (**A, B**) Cytotoxicity of EGCG (epigallocatechin-3-gallate) on 9L and M21 tumor cells. 9L (A) and M21 (B) cells were cultured in 96-well plates, then treated with increasing concentrations of EGCG ranging from 1 to 1000 µM for 2 hours. Next, cells were grown for further 48 hours without the drug. Cell survival was determined by using the MTT assay and expressed as percentage of cells counted in parallel cultures without the drug. The IC_{50} dose of EGCG determined by GraphPad Prism using nonlinear regression with variable slope was 203.7 µM for 9L cells and 361.9 µM for M21 cells. The X-axis is in the log(10) scale and the data fitted to Hill equation. (**C, D**) Determination of optimal EGCG dose in 9L and M21 cells using luciferase assay. 9L (C) and M21 (D) cells were transduced with RGD-Luc targeted vector or control fd-Luc non-targeted vector after 2 hours pretreatment with 100 µM EGCG for 9L cells and 200 µM EGCG for M21 cells. Luciferase assay was performed at day 3 post-transduction. The results were normalized to the amount of protein and presented as RLU (Relative Luminescence Units)/µg of protein. Data represent the mean ± standard error of the mean (s.e.m.) of triplicate samples.

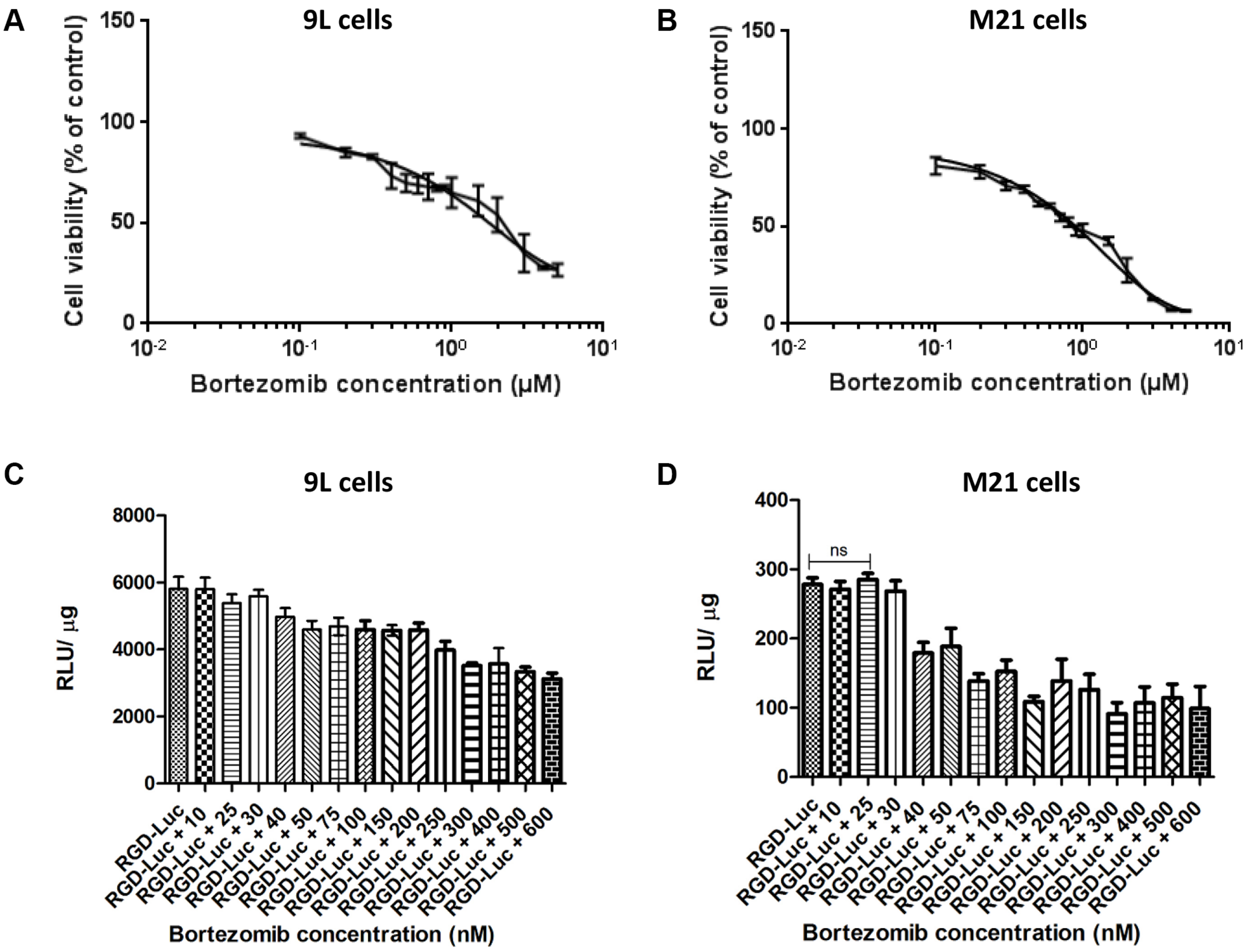

Supplementary Figure S3: (**A, B**) Cytotoxicity of bortezomib on 9L and M21 tumor cells. 9L (A) and M21 (B) cells were cultured in 96-well plates, then treated with increasing concentrations of bortezomib ranging from 0.1 to 5 μM for 2 hours. Next, cells were grown for further 48 hours without the drug. Cell survival was determined by using the MTT assay and expressed as percentage of cells counted in parallel cultures without the drug. The IC_{50} dose of bortezomib determined by GraphPad Prism using nonlinear regression was 1.55 μM for 9L cells and 1 μM for M21 cells. The X-axis is in the log(10) scale and the data fitted to Hill equation. (**C, D**) Determination of optimal bortezomib dose in 9L and M21 cells using luciferase assay. 9L (C) and M21 (D) cells were transduced with RGD-Luc targeted vector or control non-targeted vector (data not shown) after 2 hours pretreatment with increasing concentrations of bortezomib ranging from 10 nM to 600 nM. Luciferase assay was performed at day 3 post-transduction. The results were normalised to the amount of protein and presented as RLU (Relative Luminescence Units)/μg of protein. Data represent the mean ± standard error of the mean (s.e.m.) of triplicate samples.

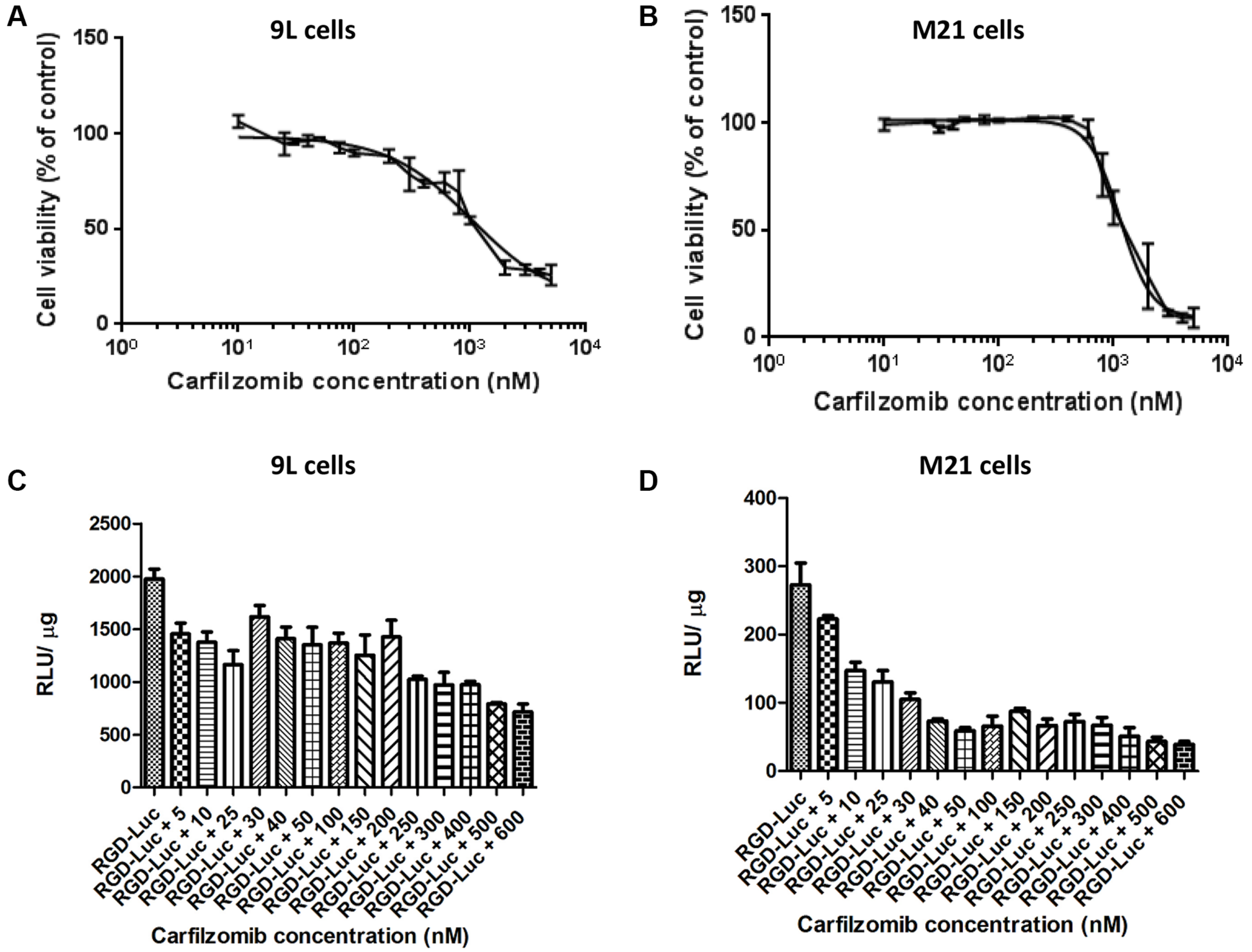

Supplementary Figure S4: (**A, B**) Cytotoxicity of carfilzomib on 9L and M21 tumor cells. 9L (A) and M21 (B) cells were cultured in 96-well plates, then treated with increasing concentrations of carfilzomib ranging from 10 to 5000 nM for 2 hours. Next, cells were grown for further 48 hours without the drug. Cell survival was determined by using the MTT assay and expressed as percentage of cells counted in parallel cultures without the drug. The IC_{50} dose of carfilzomib determined by GraphPad Prism using nonlinear regression was 1034 nM for 9L cells and 1141 nM for M21 cells. The X-axis is in the log(10) scale and the data fitted to Hill equation. (**C, D**) Determination of optimal Carfilzomib dose in 9L and M21 cells using luciferase assay. 9L (C) and M21 (D) cells were transduced with RGD-Luc targeted vector or control non-targeted vector (data not shown) after 2 hours pretreatment with different concentrations of carfilzomib ranging from 5 nM to 600 nM. Luciferase assay was performed at day 3 post-transduction. The results were normalized to the amount of protein and presented as RLU (Relative Luminescence Units)/µg of protein. Data represent the mean ± standard error of the mean (s.e.m.) of triplicate samples.
